# Efficacy of Direct Acting Antivirals (DAA) therapy in patients with recurrent hepatitis C after liver and kidney transplantation: a cross-sectional study

**DOI:** 10.3389/fmed.2024.1460372

**Published:** 2024-10-09

**Authors:** Mehdi Kashani, Mehdi Karimi, Alireza Sharifi Rayeni, Mohammad Ali Azizi Nadian, Masoud Mortezazadeh, Amirhossein Parsaei, Nooshin Abolghasemi, Niyousha Shirsalimi, Abbas Mofidi, Seyyed Taher Seyyed Mahmoudi

**Affiliations:** ^1^Department of Gastroenterology, Sina Hospital, Tehran University of Medical Sciences, Tehran, Iran; ^2^Bogomolets National Medical University (NMU), Kyiv, Ukraine; ^3^Modarres Hospital, Shahid Beheshti University of Medical Sciences, Tehran, Iran; ^4^School of Medicine, Tehran University of Medical Sciences, Tehran, Iran; ^5^Department of Internal Medicine, Sina Hospital, Tehran University of Medical Sciences, Tehran, Iran; ^6^Department of Pharmacology, Islamic Azad University - Pharmaceutical Sciences Branch, Tehran, Iran; ^7^Faculty of Medicine, Hamadan University of Medical Science (UMSHA), Hamadan, Iran; ^8^Faculty of Medicine, Tabriz University of Medical Sciences, Tabriz, Iran

**Keywords:** viral hepatitis, hepatitis C virus, HCV, direct-acting antiviral, antivirals, transplantation

## Abstract

**Background and objectives:**

Direct-acting antiviral (DAA) agents are now widely used to treat patients with hepatitis C infection (HCV) and effectively increase their sustained virologic response (SVR). However, the literature seems to lack or deficient evidence of DAA efficacy in more complicated patients, especially those with HCV reinfection after liver transplantation (LT) or liver-kidney (hepatorenal) transplantation (LKT). This study aimed to retrospectively evaluate the effectiveness of two different DAA regimens in LT and LKT patients with HCV reinfection.

**Methods:**

This cross-sectional study was conducted at three hospitals in Tehran, Iran, from 2014 to 2020, enrolling 53 patients with recurrent HCV infection after LT (*n* = 35) or LKT (*n* = 18). Patients were treated for 12 weeks with one of two DAA regimens: 37 patients (70%) received Daclatasvir and Sofosbuvir (SOF + DCV), while 16 patients (30%) received Sofosbuvir and Ledipasvir (SOF + LDV). Ribavirin (RBV) was added as an adjunct antiviral in 28 patients (52.8%). To assess the SVR, all patients were followed for 12 weeks after treatment.

**Results:**

Both DAA regimens were well-tolerated and effective, with 94.6% (35 of 37) achieving SVR-12 in the SOF + DCV group and 93.8% (15 of 16) in the SOF + LDV group. Additionally, SVR-12 rates were promising across treatment durations, with 93.9% (31 of 33) in the 12-week group and 95% (19 of 20) in the 24-week group achieving undetectable HCV RNA. No significant difference in SVR was observed between the two regimens (*p* = 0.439).

**Conclusion:**

The DAA-based therapeutic regimen was well tolerated and showed significant effectiveness in achieving the virologic response in patients with HCV reinfection after LT or LKT.

## 1 Introduction

Hepatitis C virus (HCV) infection remains a significant global health challenge, affecting millions of individuals, and is the primary cause of hepatic mortality worldwide (1, 2). HCV infection leads to severe liver diseases such as cirrhosis and hepatocellular carcinoma (HCC) (3, 4). HCV infection is a common reason for liver transplantation (LT). Persistent and recurrence of HCV infection after transplantation can cause severe graft damage and is nearly universal in patients who were viremic before the procedure (5, 6). For patients who have undergone LT, the recurrence of HCV poses a substantial risk, often leading to graft failure and reduced survival rates. Faster progression of chronic kidney disease (CKD) in HCV patients alongside higher prevalence of HCV in patients with end-stage renal disease (ESRD) on routine hemodialysis and kidney transplant recipients challenges the treatment in patients with simultaneous liver-kidney (hepatorenal) impairments (7). HCV infection after renal transplantation reduces graft acceptance and patient survival, but novel therapeutic strategies like direct-acting antiviral (DAA) agents can improve outcomes (8).

Treating HCV after transplantation is challenging, especially for kidney transplant recipients, as interferon-based therapies can increase the risk of graft rejection (9). The advent of DAA treatments has significantly improved management options for these patients (10, 11). Additionally, effective immunosuppression management is crucial for minimizing HCV reactivation risk and optimizing transplant recipients’ outcomes (12).

DAAs are novel drugs for treating HCV infection, directly targeting the HCV. Introducing these agents revolutionized the treatment of chronic HCV infection (10, 11). DAAs offer a higher rate of SVR and a lower incidence of adverse effects compared to previous interferon-based treatments (13). Sofosbuvir (SOF), a nucleotide analog inhibitor of HCV NS5B polymerase, has proven to be highly effective and well-tolerated in HCV-infected patients with cirrhosis and LT recipients with HCV reinfection (14). Recent studies suggest that SOF, in combination with other DAA agents such as Daclatasvir (DCV) and Ledipasvir (LDV), is even more effective in these patients (15). However, the search continues for the optimal DAA-based regimen and the potential benefits of combining it with other agents, such as the widely used medication ribavirin (RBV).

Although previous studies showed the desirable effectiveness of DAA agents against almost all subtypes of HCV RNA in patients with cirrhosis or renal impairment (16, 17), the evidence of LT or patients with liver-kidney (hepatorenal) transplants (LKT) due to hepatorenal syndrome is ambiguous. Few studies have tried to determine the most efficient regimen of DAA agents, the time of treatment, and its duration in patients with post-transplant HCV reinfection, especially participants with higher MELD scores and increased risk of re-transplantation (18, 19).

Given the immunosuppressive regimen required to prevent organ rejection, post-transplant patients present a distinct set of challenges, including potential drug–drug interactions and altered pharmacokinetics, which could influence the efficacy and safety of DAAs. This study aims to investigate the effectiveness of treatment with DAAs in patients with recurrent HCV following LT. By conducting a cross-sectional analysis, we seek to provide robust data on the virological response, liver function improvement, and overall patient outcomes associated with DAA therapy in this unique cohort.

## 2 Methods

### 2.1 Study design and settings

This cross-sectional study was conducted from 2014 to 2020 at three centers in Tehran, Iran, including *Sina*, *Shariati*, and *Imam Khomeini hospitals*. The study aimed to retrospectively evaluate the effectiveness of two different DAA regimens in LT and LKT patients with HCV reinfection.

### 2.2 Patients’ selection

This study included 53 adult patients with recurrent HCV infection following LT or combined LKT over a six-year period. The primary reasons for transplantation among these patients were end-stage liver failure and severe hepatorenal syndrome. Patients with HCV infection and decompensated cirrhosis, however, did not undergo organ transplantation and were therefore excluded from this study. Prior to transplantation, all included patients had confirmed HCV infection with a viral load exceeding 50 IU/mL. Patients who did not complete their treatment or follow-up, mainly due to adverse events from medications, were also excluded. Demographic and clinical data were collected from their medical records.

### 2.3 HCV therapeutic regimes

To treat HCV infection, direct-acting antivirals (DAA) were administered to patients who had undergone LT or LKT due to chronic HCV infection. The HCV genotypes were identified using Restriction Fragment Length Polymorphism (PCR-RFLP), and serum HCV RNA levels were measured by single polymerase chain reaction (PCR). HCV RNA levels below 50 IU/mL were considered undetectable and thus negative.

Based on their HCV genotypes, patients were treated for 12 weeks with one of two DAA regimens: 37 patients received DAA regimen 1 (SOF 400 mg plus LDV 90 mg), while 16 patients received DAA regimen 2 (SOF 400 mg plus DCV 60 mg). Ribavirin (RBV) was also included as an adjuvant in the antiviral regimen, and immunosuppressive therapy was continued. None of the patients had previously received DAA therapy and were therefore naive to DAA treatment prior to transplantation.

**Figure 1:**
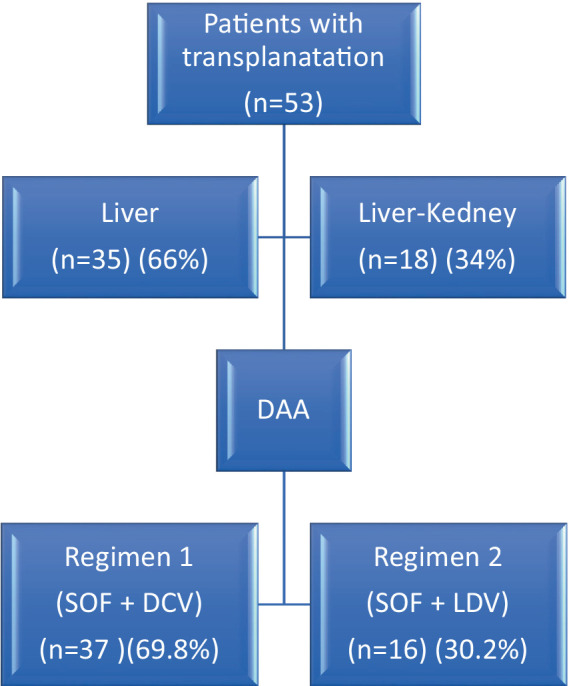
Flowchart of included patients with recurrent HCV with DAA treatment regimens.

Table 1 and Figure 1 exhibit a summary of therapeutic methods and regimes for HCV infection.

**Table 1:** Antiviral therapeutic regimes for HCV infection.

Medicine	Antivirals (dosages)	RoA	Duration	No. of patients (*n* = 53)
DAA—regimen 1	SOF (400 mg/d) + DCV (60 mg/d)	Orally	12 weeks	37 (69.8%)
DAA— regimen 2	SOF (400 mg/d) + LDV (90 mg/d)	Orally	12 weeks	16 (30.2%)
Adjuvant antiviral	RBV (1,200 mg/d)	Orally	12 weeks	28 (52.8%)

DAA, Direct-Acting Antiviral; RoA, Route of administration; SOF, Sofosbuvir; LDC, Ledipasvir; DVC, Daclatasvir; RBV, Ribavirin.

### 2.4 Immunosuppressive treatment protocol

Various immunosuppressive regimens were used to prevent transplant rejection, depending on each patient’s characteristics. Approximately half of patients (50.9%) received Mycophenolate mofetil + tacrolimus. Despite receiving these drugs, the HCV infection did not recur during treatment with antivirals (see Table 2).

**Table 2:** Treatment protocol of patients with HCV after LT or LKT.

Regimes	Medicines	Frequency
DAA	Regimen 1 (SOF + DCV)	37 (69.8%)
	Regimen 2 (SOF + LDV)	16 (30.2%)
Adjuvant antiviral	with RBV	28 (52.8%)
	without RBV	25 (47.2%)
Immunosuppressives	MFM + Cyclosporine	13 (24.5%)
	MFM + Tacrolimus	27 (50.9%)
	MFM + Sirolimus	5 (9.4%)
	MFA + Cyclosporine	4 (7.5%)
	MFA + Tacrolimus	4 (7.5%)

DAA, Direct-Acting Antivirals; MFM, Mycophenolate mofetil; MFA, Mycophenolic acid, SOF, Sofosbuvir; DVC, Daclatasvir; LDC, Ledipasvir; RBV, Ribavirin.

### 2.5 Patient assessments and follow-up

After transplantation, patients underwent HBV serological testing to determine their HBV status and detect any occult HBV infection. Then, they were treated for 12 weeks by DAA therapy, followed by an observation period during weeks 12–24. HCV RNA levels were measured every 4 weeks during 12 weeks of DAA therapy and 12 weeks of follow-up to assess sustained virologic response (SVR) to DAA therapy.

Throughout the follow-up, patients underwent routine physical examinations and various laboratory and paraclinical tests, including complete blood count (CBC), liver function tests (LFTs), and renal function tests (RFTs).

### 2.6 Ethical considerations

The study was conducted by the principles outlined in the Declaration of Helsinki. Approval for the study protocol was obtained from the Institutional Review Board (IRB) of the Tehran University of Medical Sciences (TUMS), Tehran-Iran. Participants were provided detailed information about the study, including its purpose, procedures, potential risks, and benefits. They were assured of their right to withdraw from the study without any consequences to their ongoing medical care. Informed written consent was obtained from all participants. Confidentiality and anonymity of the participants were strictly maintained throughout the study, and all data were securely stored and accessed only by authorized personnel.

### 2.7 Statistical analysis

In the study, statistical analysis was conducted to evaluate the treatment outcomes. Continuous variables were described using mean and standard deviation (SD), measuring central tendency and variability. Categorical variables were presented as counts and percentages to summarize the frequency distribution of the data. The primary efficacy endpoint was the proportion of patients achieving sustained virological response (SVR) post-treatment. A Chi-Square test was employed to compare the efficacy of two different DAA regimens in achieving SVR, assessing the statistical significance of the observed differences. All statistical analyses were performed using IBM SPSS Version 26 software, ensuring rigorous and standardized data handling and interpretation.

## 3 Results

### 3.1 Demographic characteristics of patients

Table 3 exhibits a summary of the basic characteristics of patients.

**Table 3:** Demographic characteristics of participants.

Variables		Frequency
Age	Mean (± SD)	53 (±10)
	Range	25–75
Gender	Male	37 (69.8%)
	Female	16 (30.2%)
BMI	Mean (± SD)	25.01 (±3.82)
Transplantations	Liver	35 (66%)
	Liver-kidney	18 (34%)

This study analyzed the data of 53 patients with recurrent HCV infection who had undergone LT or LKT. The average age of the participants was 53 ± 10 years, ranging from 25 to 75 years. Among the participants, 37 (69.8%) were male, and 16 (30.2%) were female. Of the participants, 35 (66%) had LT, while 18 (34%) received hepatorenal transplants. The mean Body Mass Index (BMI) was 25.01, indicating a normal weight range among the patients (see Figure 1).

The distribution of blood groups, with O+ (37.7%) and A+ (30.2%) being the most common among the patients, while the lowest rate of blood group was O^−^ (3.8%) and B^−^ (3.8%), respectively. Most participants (66%) underwent LT, while 34% received LKT. The mean HCV viral load for the patients at the beginning of the treatment was 6.00 (±1.21) log IU/mL. The majority of patients (64.2%) were infected with HCV genotypes 1a (32.1%) and 3a (32.1%), while the lowest infection rates were observed for genotypes 2a (3.8%) and 4 (5.7%) (see Table 4).

**Table 4:** Baseline laboratory data of patients.

Blood tests	Unit	Frequency (n/%)
Blood group	O **+**	20 (37.7%)
	O **–**	2 (3.8%)
	B **+**	10 (18.9%)
	B **–**	2 (3.8%)
	A **+**	16 (30.2%)
	A **–**	3 (5.7%)
RBC	10^**12**^/L	4.9 (±0.5)
WBC	10^**9**^/L	5,180 (±2017)
Platelets	10^**9**^/L	174 (±78)
Creatinine	mg/dL	1.55 (±1.19)
Serum Albumin	g/dL	3.9 (±1.1)
Total Bilirubin	mg/dL	0.55 (±0.40)
AST	IU/L	61 (±58)
ALT	IU/L	66 (±67)
ALP	IU/L	230 (±128)
MELD score	Score	18 (±3)
HCV genotype	1a	17 (32.1%)
	1b	9 (17%)
	2a	2 (3.8%)
	3a	17 (32.1%)
	3b	5 (9.4%)
	4	3 (5.7%)
HCV viral load	(log IU/mL)	6.00 (±1.21)

BMI, Body mass index; DAA, Direct-acting antivirals; SOF, Sofosbuvir; LDV, Ledipasvir; DCV, Daclatasvir; MELD, Model for End-Stage Liver Disease; RBC, red blood cells; WBC, white blood cells; Cr, creatinine; AST, aspartate aminotransferase; ALT, alanine transaminase; ALP, alkaline phosphatase.

The basic laboratory data of the patients are presented in Table 4.

### 3.2 Efficacy and comparison of DAA regimens

**Figure 2:**
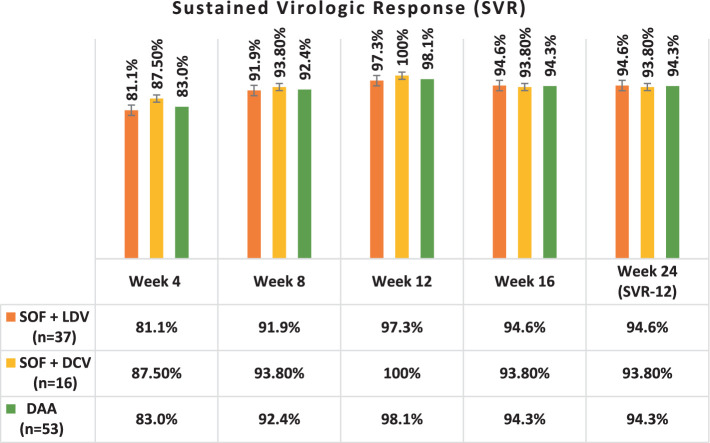
The sustained virologic response (SVR) to DAA in patients during 24 weeks of follow-up. SOF, Sofosbuvir; LDV, Ledipasvir; DCV, Daclatasvir.

Table 5 and Figure 2 present the SVR rates achieved in patients treated with two different DAA regimens over 12 weeks, followed by a 12-week follow-up phase. Both regimens demonstrated high rates of SVR throughout the treatment and follow-up periods.

**Table 5:** The sustained virologic response (SVR) to DAA in patients during 24 weeks of DAA therapy and follow-up.

Time	Weeks	SVR	Regimen 1 (SOF + DCV) (*n* = 37)	Regimen 2 (SOF + LDV) (*n* = 16)	DAA (*n* = 53)
DAA therapy	Week 4	–	81.1% (30)	87.5% (14)	83% (44)
	Week 8	–	91.9% (34)	93.8% (15)	92.4% (49)
	Week 12	–	97.3% (36)	100% (16)	98.1% (52)
Follow up	Week 16	SVR-4	94.6% (35)	93.8% (15)	94.3% (50)
	Week 24	SVR-12	94.6% (35)	93.8% (15)	94.3% (50)

SOF, Sofosbuvir; LDV, Ledipasvir; DCV, Daclatasvir; DAA, Direct-Acting Antiviral; SOF, Sofosbuvir, LDV, Ledipasvir, DCV, Daclatasvir, SVR, Sustained virologic response.

After completing a 12-week period of DAA therapy, 36 out of 37 (97.3%) patients who received the DAA regimen 1 (SOF + DCV) and all 16 (100%) patients who received regimen 2 (SOF + LDV) achieved undetectable HCV RNA levels. This resulted in the highest SVR rates observed at week 12 for both regimens, with SVR-12 rates of 97.3 and 100%, respectively.

A comparison of the two DAA regimens, regimen 1 (SOF + DCV) and regimen 2 (SOF + LDV), in achieving SVR at week 12 (SVR-12), revealed no significant difference between the groups (*p* = 0.439).

At the end of DAA therapy at the end of week 12, 52 patients had undetectable HCV RNA levels, and only one had a detectable viral load, indicating no treatment response to these DAA regimens.

In week 24, 50 patients had undetectable HCV RNA levels, and Only 3 (5.7%) patients had a detectable viral load indicating recurrent HCV infection, resulting in SVR-12 94.3% (SVR-12 = 94.3%).

There was no significant difference in SVR between LT and LKT recipients, with 16 out of 18 (88.9%) and 34 out of 35 (97.1%) achieving SVR-12, respectively. Both DAA regimens were highly effective, with 35 out of 37 (94.6%) patients on SOF + DCV and 15 out of 16 (93.8%) on SOF + LDV showing positive results.

Among the 8 patients with ESRD (GFR <15) undergoing routine hemodialysis, only one had detectable HCV RNA 12 weeks after treatment. Both the 12-week and 24-week treatment durations showed promising results, with 31 out of 33 patients (93.9%) in the 12-week group and 19 out of 20 patients (95%) in the 24-week group achieving undetectable HCV RNA levels.

### 3.3 Efficacy of ribavirin

In the study, 28 patients (52.8%) received RBV as an additional antiviral treatment alongside the DAA regimen for treating HCV to improve SVR (20). Among these patients, RBV was added to the treatment regimen of 17 patients (32%) in the regimen 1 (SOF + DCV) group and 11 patients (20.7%) in the regimen 2 (SOF + LDV) group.

The addition of RBV did not significantly impact the SVR12 rates for both treatment regimens. Without Ribavirin, 23 out of 25 patients (92%) achieved SVR12; with Ribavirin, 27 out of 28 (96.4%) achieved SVR12.

## 4 Discussion

This retrospective study aimed to investigate the efficacy of DAA-based regimens in treating patients with recurrent HCV infection after LT or LKT. The study’s findings are significant, as they indicate that DAA-based regimens showed promising efficacy in achieving SVR in these LT patients who received immunosuppressants. It also investigated the impact of adding RBV as an adjuvant antiviral on treatment duration and the proportion of patients with SVR.

This study found that two DAA-based regimens, including SOF + LDV and SOF + DCV, could effectively provide a SVR for at least 3 months in patients with recurrent HCV infection after LT or LKT, regardless of age and gender. We did not observe significant differences between participants with LT recipients and those with LKT. Additionally, adding RBV did not result in significant changes in patients with SVR. DAA agents demonstrated strong efficacy against all HCV subtypes in LT recipients with different MELD scores.

Our findings confirm the effectiveness of SOF + DCV and SOF + LDV in treating post-LT HCV reinfection, achieving SVR12 rates of 94.6 and 93.8%, respectively. Our two DAA regimens showed no significant difference in the proportion of participants, whether treatment-naive or treatment-experienced, with undetectable HCV RNA. The findings align with a randomized controlled trial (RCT) conducted by Merat et al. (21). In 2016, Fontana et al. Fontana demonstrated the efficacy of DCV + SOF or SMV in LT recipients with severe HCV recurrence, achieving an 87% SVR12 rate. Similarly, Kwok et al. (22) reported a 96% SVR12 rate in 204 post-LT HCV-infected patients treated with SOF + LDV without RBV (22).

Our current results confirm the effectiveness of SOF and DCV or SOF and LDV in treating HCV reinfection after LT, with SVR12 rates of 94.6 and 93.8%, respectively. Our study found no significant difference in the proportion of participants, whether treatment-naive or treatment-experienced, with undetectable HCV RNA when using these two DAA regimens, which is consistent with previous literature (21). A study showed that the combination of SOF and LDV without RBV was able to achieve a SVR at 12 weeks in 96% of participants (23).

HCV genotype plays a crucial role in selecting the most appropriate DAA-based regimen. In line with previous studies and evidence on HCV epidemiology (24), our results showed no significant difference in SVR12 rates between HCV genotypes 1a and 3a (88.2% for 1a and 94.1% for 3a, *p* < 0.05). However, the small sample sizes for genotypes 2a, 3b, and 4 (2, 5, and 3 patients) limited our ability to compare their outcomes.

Jacobson et al. (25) investigated the efficacy of SOF in patients with HCV genotype 2 or 3. They found that the 12-week virologic response rate for genotype 3 was lower than for genotype 2, but extending treatment to 16 weeks significantly increased SVR rates for genotype 3. In a study of 79 LT recipients with HCV reinfection, Agarwal et al. (26) reported SVR12 rates for all HCV genotypes 1 to 4 treated with a 12-week SOF + VEL regimen, achieving favorable virologic responses for each genotype (SVR12 ≥ 95%). Similarly, Feld et al. (27) observed the same results in a study of 624 patients with genotypes 1, 2, 4, 5, and 6 treated with the same regimen (27).

Researchers have recently focused on optimizing DAA-based treatment durations and identifying beneficial adjuvant medications. Pungpapong et al. (28), in a multi-center study, investigated the efficacy of DAA agents (SOF + SMV) in 123 LT recipients with HCV reinfection, with 25 (20.3%) patients also receiving RBV (28). The addition of RBV slightly enhanced the SVR12 rate, but this difference was not statistically significant. However, RBV significantly increased the incidence of anemia compared to the RBV-free regimen (28).

Flamm et al. (29) conducted a study on 108 HCV-infected patients with decompensated cirrhosis to examine the effect of treatment duration on DAA-based therapy outcomes. They divided the participants into two groups: 53 patients received SOF + LDV + RBV for 12 weeks, and 55 received the same regimen for 24 weeks. They found no significant difference in the SVR12 rate between the 12-week and 24-week treatment periods. Our study supports this finding, as neither treatment extension nor RBV addition significantly changed the proportion of patients with undetectable HCV RNA. However, our results did not show a considerable difference in adverse events for the RBV group, likely due to the high prevalence of complications like anemia or neutropenia among our patients, many of whom have other conditions, particularly renal impairments.

The impact of DAA agents on patients with more advanced liver disease (higher MELD or CTP scores) and those in “MELD purgatory”—where patients drop off the transplant waiting list due to improved MELD scores without clinical advancements—remains controversial (30). Few studies have investigated the effectiveness of DAAs in post-transplant patients with severe cirrhosis due to HCV reinfection who are at risk for re-transplantation. Previous research has demonstrated the significant efficacy of DAAs in patients with advanced liver cirrhosis. For instance, Fontana et al. (31) found substantial MELD score improvements in LT recipients with baseline MELD≥15 using both DCV + SMV ± RBV and DCV + SOF ± RBV regimens. Conversely, Pellicelli et al. (32) did not observe considerable improvements in their study on 12 post-transplant patients with recurrent HCV infection. They advised against using DAAs in advanced liver disease due to minimal clinical benefits, advocating for early-stage treatment instead (32).

A study conducted by Sasso et al. (33) found that switching stable liver transplant patients from a twice-daily (BID) tacrolimus regimen to a once-daily (QD) formulation was effective and safe. The conversion did not significantly alter tacrolimus levels or daily doses and maintained stable liver and metabolic parameters. Renal function improved post-conversion, and no acute rejection or major adverse events occurred. Additionally, patient-reported outcomes indicated better adherence to the immunosuppressive regimen. This switch potentially enhances the quality of medical care for liver transplant recipients in real-world settings (33).

Valente et al. (34), in a multicenter study in 2021, found that treating HCV-positive prediabetic patients with DAAs significantly reduces the incidence of major cardiovascular events (MACEs) compared to untreated controls. Their results indicated that HCV eradication through DAAs is associated with a lower rate of cardiovascular events, making it a crucial treatment goal for improving cardiovascular outcomes in prediabetic patients, irrespective of liver disease severity or other cardiovascular risk factors (34).

Our study found significant improvements in MELD scores and virologic and clinical responses to DAA-based therapy. Among eight patients with MELD>20, only one—a 65-year-old female intravenous drug user—failed to achieve SVR12. Given the high risk of re-transplantation surgery for patients with multiple comorbidities, DAA-based regimens (SOF + DCV or SOF + LDV) demonstrated excellent virologic responses and acceptable clinical improvements in high-risk liver re-transplant candidates.

### 4.1 Limitations of study

One limitation of this study was the small sample size of LT and LKT recipients with recurrent HCV infection, which limited our ability to thoroughly compare variables that could influence their response to DAA-based treatment. Additionally, the presence of multiple comorbidities and concurrent medications among patients made it challenging to assess DAA adverse events, potentially introducing bias accurately. Another limitation was our inability to measure SOF, DCV, or LDV serum levels, which could have offered valuable insights into the pharmacokinetics and potential dosage adjustments needed for LT recipients. Although it would have been ideal to follow up with patients 24 weeks after the end of treatment, logistical constraints prevented this; we recommend this for future studies.

### 4.2 Strength of study

A notable strength of this study is its focus on a highly specific and clinically significant population—LT recipients with recurrent HCV infection. By concentrating on this group, the study effectively highlights the real-world challenges and outcomes associated with DAA-based treatment in a complex patient cohort. This focus provides critical insights into the efficacy and safety of DAAs in a context that is often underrepresented in clinical research. Additionally, the study underscores the need for further prospective, multi-regional cohort studies to deepen our understanding of DAA therapy’s effectiveness and safety across diverse populations. Such research could lead to more tailored and effective treatment strategies, offering a ray of hope and ultimately improving the management of HCV in transplant recipients and contributing significantly to the field of hepatology.

## 5 Conclusion

In conclusion, DAA treatment effectively produced a virologic response in LT recipients with HCV reinfection. The DAA agents were valuable in treating these patients due to their ability to improve hepatic function, high tolerability, and low incidence of adverse events. This was especially true for patients with simultaneous LKT or those at risk of re-transplantation due to high-grade cirrhosis.

## Data Availability

The original contributions presented in the study are included in the article/supplementary material, further inquiries can be directed to the corresponding authors.
